# Testing the Analytical Rumination Hypothesis: Exploring the Longitudinal Effects of Problem Solving Analysis on Depression

**DOI:** 10.3389/fpsyg.2020.01344

**Published:** 2020-07-02

**Authors:** Marcela Sevcikova, Marta M. Maslej, Jiri Stipl, Paul W. Andrews, Martin Pastrnak, Gabriela Vechetova, Magda Bartoskova, Marek Preiss

**Affiliations:** ^1^National Institute of Mental Health, Klecany, Czechia; ^2^First Faculty of Medicine, Charles University, Prague, Czechia; ^3^Krembil Centre for Neuroinformatics, Centre for Addiction and Mental Health, Toronto, ON, Canada; ^4^Department of Psychology, Neuroscience and Behaviour, McMaster University, Hamilton, ON, Canada; ^5^Third Faculty of Medicine, Charles University, Prague, Czechia; ^6^Department of Psychology, Faculty of Education, Charles University, Prague, Czechia; ^7^University of New York in Prague, Prague, Czechia

**Keywords:** depression, analysis, evolution, analytical rumination hypothesis, problem-solving

## Abstract

Depression is a mental health condition for which individuals commonly seek treatment. However, depressive episodes often resolve on their own, even without treatment. One evolutionary perspective, the analytical rumination hypothesis (ARH), suggests that depression occurs in response to complex problems. According to this perspective, depressive symptoms promote analytical rumination, i.e., distraction-resistant thoughts about the causes of problems [causal analysis (CA)] and how they can be solved [problem-solving analysis (PSA)]. By helping individuals solve complex problems, analytical rumination may contribute to remission from depression. The aim of this study was to investigate (1) whether clinically-depressed individuals have more complex problems and engage in more CA and PSA than non-depressed and (2) the effects of CA and PSA on decreases in problem complexity, depressive symptoms, and remission from the depression. Samples of 85 patients were treated for depression with antidepressants and psychotherapy, and 49 healthy subjects were assessed three times over a 4-month period (at Weeks 1, 5, and 16). At each assessment, they completed measures of depression, analytical rumination, and problem complexity. Depressed individuals reported having more complex problems and engaging in more CA than non-depressed participants. The two groups engaged in a similar degree of PSA. Findings from a multiple regression suggested that more PSA at Week 1 was related to a decrease in depressive symptoms at Week 5, even after controlling for baseline depression, problem number, and complexity. PSA at Week 1 did not predict the remission after hospitalization or at follow-up; however, having less complex problems at the baseline made it more likely that a patient would later remit. Engaging in more CA or PSA at Week 1 did not affect perceived problem complexity at Week 5 or at follow-up. However, these findings were not statistically significant when influential observations (or outliers) were included in the analysis. Our findings suggest that PSA may contribute to a decrease in symptoms of depression over time. However, alleviations in problem complexity and remission might only be achieved if problems are initially less complex. Future directions involve exploring how PSA might contribute to decreases in depressive symptoms and other mechanisms underlying remission from depression.

## Introduction

Major depressive disorder (MDD) is a common mental health condition (World Health Organization, 2017) and one of the leading causes of disability worldwide (Friedrich, 2017). It is primarily characterized by persistent sadness and a loss of interest that interferes with daily functioning. Other symptoms, such as changes in activity, sleeping and eating patterns, fatigue, a diminished ability to think or concentrate, suicidal thoughts, and rumination (i.e., intense, persistent thinking about a depressive episode), might also be present (American Psychiatric Association, 2013). Given that MDD is a highly prevalent and burdensome condition, it is important to develop our understanding of its underlying mechanisms and the remission of its symptoms.

One puzzling feature of MDD is that depressive episodes tend to resolve on their own, i.e., without treatment (Posternak and Miller, 2001; Whiteford et al., 2013). Research indicates that 20–35% of improvement in clinical trials is due to spontaneous remission (Krogsbøll et al., 2009) that about 50% of untreated depressive episodes will remit within a year (Whiteford et al., 2013). These studies suggest that regardless of whether an individual is treated for depression (e.g., with cognitive-behavioral therapy), there may be other factors that contribute to remission from MDD (Saragoussi et al., 2017; Vitriol et al., 2018). Alternative perspectives on the etiology of depression can provide insight into these factors (Durisko et al., 2016).

### Depression as a Reaction to Adversity

The etiology of MDD is usually attributed to a combined influence of stressful events and an individual’s cognitive predisposition (Lewinsohn et al., 2001). Research suggests that life stressors tend to precede or coincide with depressive episodes (Keller et al., 2007). Although this view is widely accepted, there is an ongoing debate about the nature of the predisposition and its contribution to the etiology of MDD.

Cognitive perspectives on MDD suggest that a cognitive vulnerability, namely the thoughts and attitudes following life stressors, modulates an individual’s risk of becoming depressed (Beck, 1976). Specifically, these cognitions are thought to involve a tendency to engage in negative and self-relevant thinking (Haaga et al., 1991) or an attitude of hopelessness (i.e., expectations that negative events are inevitable, uncontrollable, and have global and enduring consequences; Abramson et al., 1989). With severe MDD, these cognitions become intense and persistent, leading to a style of thinking referred to as depressive rumination (Beck, 1976). Because many studies find that individuals who are predisposed to ruminate tend to become depressed, this style of thinking is generally thought to be dysfunctional and implicated in the development of MDD (Moberly and Watkins, 2008; Nolen-Hoeksema et al., 2008). Moreover, although ruminating can sometimes be useful for gaining insight into problems and emotions (Lyubomirsky and Nolen-Hoeksema, 1993), rumination in the presence of severe MDD has been proposed to interfere with the ability to think clearly and to solve problems (Nolen-Hoeksema, 1991; Lyubomirsky and Nolen-Hoeksema, 1995). This clouded judgment and impaired problem-solving might exacerbate existing stressors and worsen MDD.

According to some evolutionary psychologists, however, depression (including its cognitive features) is an adaptation shaped by natural selection. One of the basic premises of adaptationism is that organisms are designed to produce appropriate responses to environmental triggers (Fleagle, 2013). Sadness or low mood has evolved to help animals cope with fitness threats by coordinating their physiology, cognition, and behavior (Durisko et al., 2015). Sadness is hypothesized to be aroused in response to environmental circumstances that decrease fitness, such as illness, social conflict or rejection, and a loss of status of resources (Nesse, 1990; Levenson, 1999). The subjective experience of sadness is unpleasant, which motivates individuals to think or act in ways that avoid or resolve these situations. In the context of depression then, symptoms such as sadness, anhedonia, changes in psychomotor functions, sleeping, eating and cognitive patterns may be elicited by specific triggers, and this may help an individual respond to these triggers (Nesse, 2000; Nettle, 2004; Keller and Nesse, 2006; Hagen, 2011; Durisko et al., 2015). Although several adaptationist hypotheses have been devised to explain the function of depression (see Durisko et al., 2015), most of them have not yet been tested in humans let alone in clinically depressed participants. In this study, we investigate one adaptationist hypothesis that focuses on the cognitive features of MDD, the analytical rumination hypothesis (ARH; Andrews and Thomson, 2009).

### The Analytical Rumination Hypothesis

The ARH proposes that depression evolved as a response to adverse and analytically difficult problems that recurred in the evolutionary past (reviewed in Andrews and Thomson, 2009). These problems have different sources, such as sexual infidelity or migration, but they share in common their complexity. Often, they involve dilemma-type situations with no clear solutions, and they are most often of a social nature, involving uncertain outcomes and multiple goals (e.g., to maintain cooperative bonds while pursuing self-interest). Problems are more complex if there are more possible strategies to choose from and if the strategies are in conflict with one another (Andrews and Thomson, 2009).

According to the ARH, MDD’s crucial adaptive trait is analytical rumination, which functions to resolve the complex problems triggering the depressive episode (Andrews and Thomson, 2009). In contrast to the view of rumination in MDD as a negative and unproductive style of thinking (Nolen-Hoeksema et al., 2008), Andrews and Thomson (2009) posit that rumination is useful for helping individuals address important problems in their lives. They highlight its analytical nature, suggesting that individuals with MDD engage in a process of analysis to identify the causes of their problems and find optimal solutions. In turn, finding effective ways to address the triggering problems is hypothesized to alleviate depressive symptoms, eventually leading to remission from MDD (Andrews and Thomson, 2009; Bartoskova et al., 2018). Although analytical rumination is effortful and prolonged, it may therefore be a naturally self-limiting process that can both reduce the complexity of personal problems and promote remission from depression. In other words, once the problems that triggered the depressive episode have been addressed through analytical rumination, analytical rumination and the underlying depressive episode might alleviate on its own (i.e., without medical or psychotherapeutic treatment).

### Empirical Research on the ARH

To facilitate empirical research on analytical rumination, Barbic et al. (2014) developed a tool for measuring rumination based on analysis. The resulting Analytical Rumination Questionnaire (ARQ) is psychometrically robust and clinically useful for assessing changes in analytical rumination (Barbic et al., 2014). Bartoskova et al. (2018) explored the psychometric properties of the ARQ in five samples (total *N* = 1,414) from two cultures (Canada, Czech Republic) differing in clinical status (psychiatric patients, subclinical respondents). The results of this cross-sectional study demonstrated that analytical rumination consists of two factors. The first factor, causal analysis (CA), involves attempts to understand the cause of problems or how they could have been avoided. The second factor, problem-solving analysis (PSA), focuses on finding ways to address complex problems (i.e., while navigating competing demands, difficult or constrained conditions, etc.) or taking measures to prevent similar problems in the future.

Bartoskova et al. (2018) compared the ARQ with another widely-used measure of rumination, the Ruminative Response Style Questionnaire (Nolen-Hoeksema, 1991). Items from this questionnaire similarly reflect two factors: brooding (i.e., a passive comparison with an unachieved standard) and reflective pondering (i.e., a purposeful turning inward to engage in cognitive problem-solving to alleviate depression; Treynor et al., 2003). According to their findings, these two factors (as measured with the Ruminative Response Style Questionnaire) lacked face validity, were highly correlated, and were not well differentiated (Bartoskova et al., 2018). The researchers concluded that the ARQ is better-equipped to test the ARH’s claim that individuals with MDD engage in a process of analysis to identify the causes of problems and find solutions (Andrews and Thomson, 2009).

**Figure 1:**
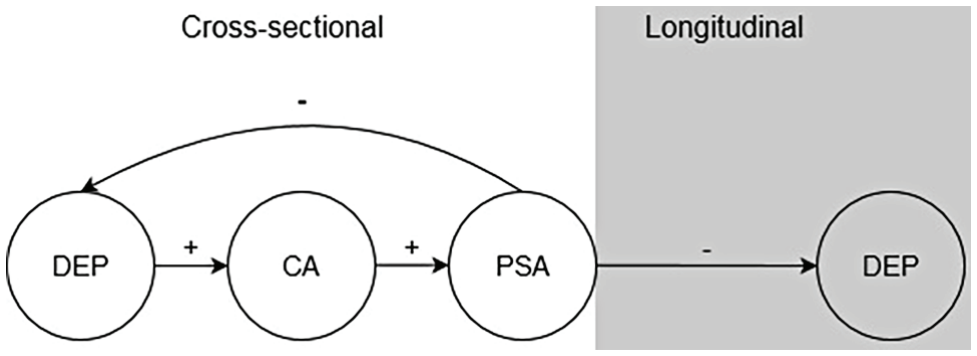
Depression (DEP) promotes causal analysis (CA), which promotes problem-solving analysis (PSA). In the short-term, PSA reduces DEP. PSA is hypothesized to reduce DEP in the long-term.

Bartoskova et al. (2018) additionally examined whether analytical rumination (as measured with the ARQ) functions to solve triggering problems. According to findings from structural equation models tested in each cross-sectional sample, depression promoted CA, which promoted PSA. Importantly, PSA decreased depression in a negative feedback pathway, suggesting that the covariance patterns between depression and PSA are complex: PSA is positively related to depression through its positive relation with CA, but its direct effect on depression is negative. These findings suggest that PSA decreases depressive symptoms and that over time, the process of CA and PSA may naturally promote recovery from MDD (see Figure 1).

**Table 1:** Descriptive sample information at each week.

	Week 1		Week 5		Week 16	
	*M* (*SD*)		*M* (*SD*)		*M* (*SD*)	
	Depressed	Non-depressed	Depressed	Non-depressed	Depressed	Non-depressed
N (N women)	85 (58)	49 (34)	67 (44)	49 (29)	51 (29)	36 (24)
Comparison	chi(1) = 0.025, *p* = 0.874					
Age	45.07 (11.84)	41.76 (12.79)	45.09 (10.92)	41.38 (12.94)	44.36 (11.62)	40.16 (12.92)
Comparison	*t*(139) = −1.752, *p* = 0.08					

N = number of participants in the respective condition.

In another set of studies on the ARH, Maslej et al. (2019) used a writing paradigm that reliably increased sadness (i.e., the emotional symptom of depression) to investigate the temporal order of emotion, CA, and PSA. Sadness during writing coincided with CA (as reported on the ARQ and supported by linguistic evidence from the writing tasks) but not PSA. This finding suggests that depressive emotion promotes CA, providing experimental support for the first stage in the process of analytical rumination (Maslej et al., 2019). Although the writing task instructed participants to write about their most important personal problems, Maslej et al. (2019) did not measure the complexity of these problems to determine how complexity was related to sadness and analytical rumination.

Thus, the cross-sectional and experimental studies conducted on the ARH to date have not addressed whether analytical rumination is related to the complexity of problems and whether it reduces the perceived complexity of problems in the long term. Similarly, it remains unclear whether effective problem-solving can occur in the presence of clinical depression or the long-term implications of this problem-solving on MDD and remission. To distinguish between the cognitive and evolutionary perspectives then, it is necessary to determine whether analytical rumination forms part of a functional process that contributes to decreases in MDD symptoms, the remission of MDD, and reduced complexity of problems over time.

### The Present Study

Although there is a well-established link between depression and life stressors (Keller et al., 2007), it is unclear whether depressed individuals perceive their problems to be complex. More empirical research is also needed to support the claim that analytical rumination forms part of an adaptive response to complex problems in patients with MDD. Specifically, it is important to test (1) whether individuals with MDD have more complex problems and engage in more analytical rumination than non-depressed individuals and (2) whether analytical rumination contributes to decrease in depressive symptoms, remission from depression, and a decrease in problem complexity over time.

To address these two issues, we recruited two groups of individuals (i.e., depressed and non-depressed) into a study. We assessed their personal problems (i.e., number and complexity), their levels of depression, and analytical rumination at multiple time points. For our depressed group, we focused on severely depressed individuals, so that we could adequately evaluate claims that severe depression prevented individuals from engaging in effective problem-solving (Lyubomirsky and Nolen-Hoeksema, 1995). Because severely depressed individuals tend to be hospitalized, our depressed group included hospitalized inpatients diagnosed with MDD. These patients were assessed in the first week and last week of their psychiatric hospitalization, as well as 16 weeks after the baseline testing (at follow-up). A second group of healthy individuals (i.e., the non-depressed group), matched on age, sex, and education, completed the same assessments at the same times as the depressed patients.

**Figure 2:**
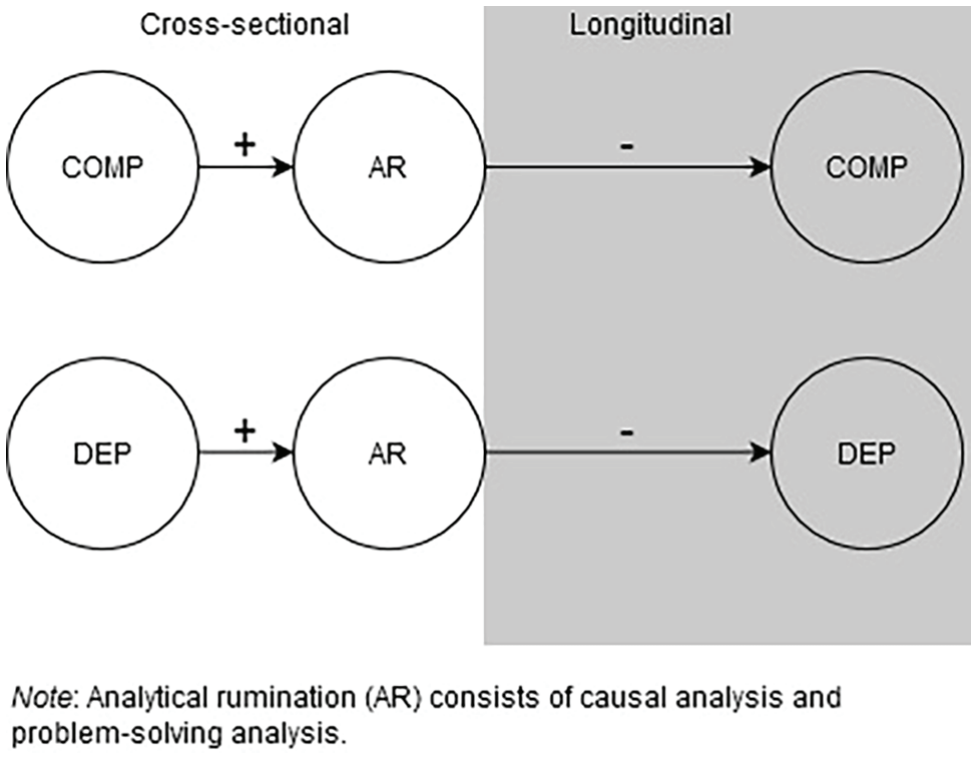
Analytical rumination (AR) is hypothesized to be promoted by complex problems (COMP) and depression (DEP). AR is predicted to decrease both COMP and DEP.

To address our first question of whether individuals with MDD have more complex problems and engage in more CA and PSA than non-depressed individuals, we conducted cross-sectional comparisons of our two groups at each time point. Based on a previous work (Andrews and Thomson, 2009), we predicted that patients with MDD would report having more problems as compared to non-depressed individuals and that they would perceive these problems to be more complex. We additionally expected that MDD patients would report engaging in more CA and PSA than non-depressed individuals, at least at the start of their hospitalization (see Figure 2).

**Table 2:** Descriptive information for depression, CA, PSA, problem-related variables, and remission at each week.

	Week 1		Week 5		Week 16	
	*M* (*SD*)		*M* (*SD*)		*M* (*SD*)	
	Depressed	Non-depressed	Depressed	Non-depressed	Depressed	Non-depressed
Depression	27.07 (7.17)	1.90 (2.45)	20.22 (9.57)	2.21 (2.10)	18.22 (11.76)	2.24 (2.96)
CA	8.27 (2.25)	5.84 (2.09)	7.49 (2.04)	5.98 (2.05)	6.88 (2.22)	6.53 (2.93)
PSA	6.74 (2.17)	6.57 (1.93)	6.90 (1.97)	6.76 (1.97)	6.75 (1.96)	7.42 (2.43)
PCQ	26.10 (4.99)	14.42 (5.12)	24.31 (5.53)	14.98 (6.17)	22.82 (6.65)	13.38 (4.98)
Problem num	4.95 (2.61)	1.37 (1.09)	3.92 (2.16)	1.29 (1.07)	3.78 (2.60)	1 (1.08)
Remitters	-	-	*n* = 16 (24%)	-	*n* = 14 (27%)	-
Non-remitters	-	-	*n* = 51 (76%)	-	*n* = 37 (73%)	-

CA, causal analysis; PSA, problem-solving analysis; PCQ, problem complexity questionnaire; Problem num, number of problems reported.

To address our second question of whether analytical rumination contributed to our outcomes of interest (i.e., decreased depressive symptoms, remission from depression, and decreased problem complexity), we conducted a longitudinal analysis in depressed patients only. Given that an estimated 20–35% of individuals remit in a period of 4–20 weeks (Posternak and Miller, 2001; Whiteford et al., 2013), we expected that some of these patients would remit by the end of their hospitalization, while others might remit after a longer time period (i.e., at follow-up). Thus, we examined the role of analytical rumination at the beginning of hospitalization on decreases in depressive symptoms, the likelihood of remission, and perceived problem complexity at the end of the hospitalization and at the 16-week follow-up (for the depressed group only). The ARH predicts complex relationships between CA, PSA, and depression (Andrews and Thomson, 2009; Barbic et al., 2014; Bartoskova et al., 2018; Maslej et al., 2019). Based on a previous work, CA is not predicted to have a direct, negative influence on depressive symptoms (Bartoskova et al., 2018). However, if individuals develop a better understanding of the nature and cause of their problems through CA, we predicted that CA might negatively predict problem complexity after hospitalization. Furthermore, if PSA helps individuals address their problems (either by generating key insights or promoting constructive action), we expected that PSA would be a negative predictor of problem complexity. Finally, we predicted that PSA would have a direct, negative influence on depressive symptoms after hospitalization and at follow-up, and eventually, a positive influence on remission from MDD (see Figure 2).

## Materials and Methods

### Participants

Two groups of adult participants (aged 18–65) were recruited into the study. The first group consisted of currently depressed inpatients at the National Institute of Mental Health hospital in Klecany, Czech Republic. These participants were recruited from the hospital’s Department of Affective Disorders over a 2-year period (from 2017 to 2018). All patients had a primary diagnosis of MDD confirmed by ward psychiatrists. At screening (referred to in the study as Week 0), this diagnosis was also verified with the “the Mini-International Neuropsychiatric Interview (M.I.N.I.),” a structured interview developed to objectively assess for the presence of psychiatric disorders (Sheehan et al., 1997). As a further inclusion criterion, this group only included patients with a depression severity score of 20 or higher (indicating moderate to severe depression), as measured using the Montgomery-Åsberg Depression Rating Scale (MADRS) during screening (Montgomery and Åsberg, 1979).

The exclusion criteria were current or past psychosis, substance abuse 12 months prior to study initiation or during the follow-up period, dementia, developmental disabilities, severe physical or neurological conditions, current pregnancy, and thyroid disorders. MDD patients were receiving treatment as usual, including psychosocial interventions (such as group therapy, art therapy, or ergotherapy). According to medical records, all of the MDD patients were receiving antidepressants and/or benzodiazepine drugs. Since the sample size was not large, we did not control for medication.

A total of 103 inpatients were invited to take part; however, only 85 of these inpatients met inclusion criteria and were enrolled into the study. There were 18 patients who were excluded: six patients were excluded due to a MADRS score < 20, one patient did not present with depression according to the M.I.N.I., one patient had somatic health complications, four patients had a change in due treatment (application of electroconvulsive therapy), and six patients withdrew from the study.

The second group consisted of 49 participants from the general (non-clinical) population approximately matched by gender, age, and education with the depressed group (see Table 1). These participants were recruited among the general population *via* an online advertisement. To be included for the study, participants had to have no prior psychiatric or neurological medical history. The second group had the same exclusion criteria as the depressed group.

At the beginning of the study at Week 1, these groups were not significantly different in terms of sex [*χ*^2^(1) = 0.025, *p* = 0.874], age [*t*(139) = −1.752, *p* = 0.08] (see Table 1), and education [*χ*^2^(142) = 2.41, *p* = 0.661] with around 5% of the sample having primary education, 58% having secondary education, and 37% having college degree. Importantly, depressed patients and healthy participants differed in the severity of their depressive symptoms (as assessed by the MADRS) at Week 1 (*W* = 49.5, *p* < 0.01), Week 5 (*W* = 60.5, *p* < 0.01), and Week 16 (*W* = 146, *p* < 0.01) (see Table 2 for means and standard deviations).

### Measures and Tools

Montgomery-Åsberg Depression Rating Scale (MADRS; Montgomery and Åsberg, 1979). The MADRS is a semi-structured clinical interview developed to objectively assess the severity of depressive symptoms. It is a gold standard measure of depressive symptoms for clinical trials. It consists of 10 items, each scored 0–6. The cut-off score for moderate depression is 20, and the cut-off score for remission is 10 (Zimmerman et al., 2004). Internal consistency of the MADRS has been shown to be high (Cronbach’s *α* = 0.85) (Hermens et al., 2006).

The Analytical Rumination Questionnaire (ARQ; Barbic et al., 2014). The ARQ measures the extent to which participants have thought about their personal problems over the past 2 weeks (e.g., the nature and cause of the problems, potential solutions, and their consequences). This questionnaire consists of 20 items scored on a 4-point Likert scale, ranging from 1 (*never*) to 4 (*always*). Internal consistency for the ARQ has been shown to be high (Cronbach’s *α* = 0.91) (Barbic et al., 2014). Based on recommendations for using the ARQ in research derived from a psychometric analysis (Bartoskova et al., 2018), we used a 6-item version to measure the analytical rumination subtypes, CA and PSA. Three items corresponding to CA were summed to generate CA scores, and three items corresponding to PSA were summed to generate a PSA score. Internal consistency for CA is 0.82 (high) and for PSA it is 0.75 (good). Each ranged from 3 to 12, with higher scores indicating more of the analytical rumination subtype (Bartoskova et al., 2018).

#### Current Problems Inventory

To assess the number of problems experienced by participants, we developed an inventory to assess problems or stressors in 14 different domains: financial, academic, employment-related, romantic relationship troubles, relationship dissolution, sickness, dilemma with no correct solution, mistake or wrong decision, important personal failure, and the threat of something bad happening in the near future. Participants were also provided with the following options: “Something happened that was not listed here” and “I am facing a problem, but I would rather keep it a secret” and “I was not facing any problems during these 2 weeks.” Participants were asked to mark a problem in any domain that they faced in the last 2 weeks, with scores ranging from 0 to 14.

Problem Complexity Questionnaire (PCQ; Durisko et al., unpublished). We assessed the perceived complexity of current problems with the PCQ. The PCQ is a self-reported questionnaire that gauges how difficult or complex participants consider their problems to be over the past 2 weeks. It consists of eight items, such as “I do not yet know how to resolve these problems” or “These problems have left me in a dilemma” (See Supplementary Section 3 for items). Each item is rated on a 4-point Likert-type scale from 1 (*completely disagree*) to 4 (*completely agree*). Scores range from 8 to 32, with higher scores indicating higher perceptions of problem complexity. Psychometric tests of the PCQ, carried out on the current sample, are described in Supplementary Section 3. Inter-item correlations range from 0.66 to 0.75, with an acceptable mean inter-item correlation (i.e., 0.69; Cristobal et al., 2007). The internal consistency is excellent (Cronbach’s *α* = 0.95) (George and Mallery, 2003).

### Procedure

After consultation with a ward psychiatrist, depressed patients who wished to take part in the study were contacted for a screening interview and a verification of their current clinical status was carried out with the M.I.N.I, the MADRS, and an exclusion criteria checklist. Screening took place during the first 3 days of their ward stay (referred to as Week 0). After the screening interview, individuals eligible for inclusion in the depressed group completed the first study assessment (i.e., the current problems inventory, PCQ, and ARQ) in the first week of their hospital treatment (Week 1). In their final week of hospital treatment (at Week 5), patients completed a second assessment and were assessed with the MADRS a second time. Finally, a third assessment with the MADRS and other measures was administered at follow-up (at Week 16). Control participants were also assessed in-person. They completed the same screening interview and assessments at the same time points as the depressed group (i.e., Weeks 0, 1, 5, and 16). All participants provided written informed consent to take part in the study and the study was approved by the National Institute of Mental Health Research Ethics Board. Participation for both groups was completely voluntary and participants received 500 CZK (22 USD) as compensation for completing the study.

### Statistical Analyses

#### Preliminary Analyses

We conducted preliminary analyses to examine depressive symptoms, problem number, and perceived problem complexity over time. In our preliminary analyses, we also examined zero-order correlations between the outcomes of interest. CA, PSA, problem number, and PCQ scores were not normally distributed; therefore, we used non-parametric hypothesis tests.

First, we used Wilcoxon signed-rank tests (non-parametric equivalents of dependent samples *t*-tests, denoted with *V*) to examine changes in depression, problem number, and perceived problem complexity over Weeks 1, 5, and 16 in depressed patients only.

Next, we conducted zero-order correlation analyses to examine associations between our outcomes of interest. To examine whether analytical rumination increased as a function of problem complexity at baseline, we examined associations between CA, PSA, and problem complexity at Week 1. We coded remitters (as 1) and non-remitters (as 0) at Weeks 5 and 16 using a cut-off score of 10 on the MADRS. We generated Spearman correlations between CA and PSA (at Week 1) and depressive symptoms, remission (as a binary variable), and problem complexity (at Weeks 5 and 16) in the depressed group only. In these correlations, we adjusted for baseline depression severity (and baseline problem complexity) by subtracting Week 1 depression (and complexity) scores from scores at Weeks 5 and 16. We also examined correlations between problem number and complexity and our outcomes of interest (reported in Supplementary Section 3). We keep the preliminary analyses separated in order to provide exact answer to the following questions:

1. *Do depressed individuals have more complex problems and do they engage in more analytical rumination than non-depressed individuals?* To address our first question, we compared the two groups on problem number and complexity, CA and PSA at Weeks 1, 5, and 16. We used Wilcoxon-rank sum tests, non-parametric equivalents of the *t*-test (denoted with W).
2. *Does analytical rumination decrease depression and problem complexity over time?* To address our second question, we investigated the role of CA and PSA in the process of recovery from depression in our sample of depressed patients only. We conducted a multiple linear regression in the depressed group to determine whether CA and PSA at Week 1 were unique predictors of depressive symptoms at Week 5 (controlling for Week 1 depression, problem number, and problem complexity). We examined unstandardized multiple regression coefficients (denoted with β) and their corresponding standard errors (SE) to identify significant predictors. To examine the effects of CA and PSA on the likelihood of remission after hospitalization, we conducted a generalized linear model (using a binomial family function), specifying Week 1 CA, PSA, depression, problem number, and problem complexity as predictors and remission as the binary dependent variable. We additionally examined the effect of CA and PSA on perceived problem complexity after hospitalization with a multiple linear regression. We specified Week 1 CA, PSA, depression, problem number, and problem complexity as predictors and Week 5 problem complexity as the dependent variable. Finally, we conducted similar linear models to investigate the role of CA and PSA at Week 1 on depressive symptoms, the probability of remission, and problem complexity at Week 16 (controlling for Week 1 depression, problem number, and problem complexity).

We conducted diagnostic tests for each model by plotting studentized residuals, and by testing whether error variances were constant and independent. To determine the presence of outliers or influential observations, we examined QQ and influence plots (provided in Supplementary Section 1). Influential values were *a priori* identified as any observations that were highlighted in these plots, based on Cook’s distance values (Cook, 1977). We repeated our multiple regression models excluding any influential observations, which we reported as our primary results. However, we provide full results of models including influential observations in Supplementary Section 2. All statistical analyses were conducted in R statistical software, Version 3.5.1. We used the *coin* package for non-parametric testing (Hothorn et al., 2008) and the *car* and *MASS* packages for diagnostic testing (Fox et al., 2007; Ripley et al., 2013). Our data and code are openly available online (Open Science Framework, 2020a,b).

## Results

### Preliminary Analyses

#### Changes Over Time

##### Changes in Depression

**Figure 3:**
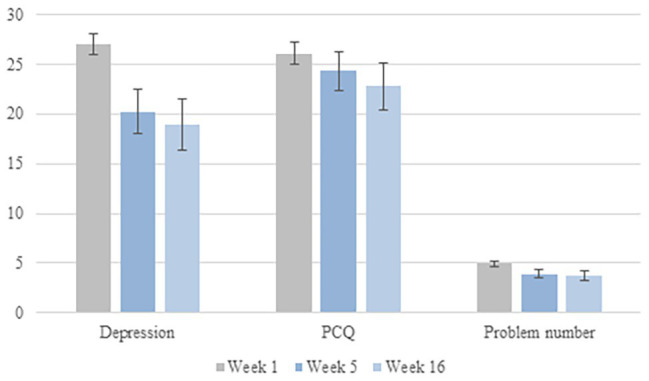
Depression, problem complexity, and problem number at each week. PCQ, problem complexity questionnaire. Error bars are within-subject 95% confidence intervals (Cousineau, 2005).

In depressed patients, the severity of symptoms decreased from Week 1 to Week 5 (*V* = 1930, *p* < 0.01) but not from Week 5 to Week 16 (*V* = 648.5, *p* = 0.92). At Week 16, patients remained less depressed than they were at Week 1 (*V* = 1092.5, *p* < 0.01) (see Figure 3).

##### Changes in Complex Problems

Depressed patients perceived their problems to be less complex at Week 5 than at Week 1 (*V* = 1,368, *p* < 0.01). However, perceived problem complexity did not change from Week 5 to Week 16 (*V* = 610.5, *p* = 0.30), so perceived problem complexity remained lower at Week 16 than Week 1 (*V* = 675, *p* < 0.01). Depressed patients also reported having fewer problems at Week 5 than Week 1 (*V* = 1097.5, *p* < 0.01), but the number of problems did not change between Weeks 5 and 16 (*V* = 433.5, *p* = 0.54). At Week 16, patients reported having less problems than they did at Week 1 (*V* = 768.5, *p* < 0.01) (see Figure 3).

#### Zero-Order Correlations

##### Associations Between CA, PSA, and Problem Complexity

At Week 1, depressed patients who perceived their problems to be more complex reported engaging in more CA, but this effect was not statistically significant, *r_s_* = 0.19, *p* = 0.08. Problem complexity was not associated with PSA, *r_s_* = −0.05, *p* = 0.64.

##### Associations Between CA and PSA (Week 1) and Changes in Depressive Symptoms, Remission, and Perceived Problem Complexity (Weeks 5 and 16)

Table 3 presents Spearman correlations between CA and PSA (at Week 1) and depression, remission, and problem complexity (at Weeks 5 and 16). Depressive symptoms at Week 5 were negatively related to PSA at Week 1, with more PSA predicting larger decreases in future symptoms. However, depressive symptoms at Week 5 were not associated with CA at Week 1. Remission at Week 5 was not associated with CA, PSA at Week 1. Finally, problem complexity at Week 5 was not associated with CA or PSA at Week 1 (Table 3). Depressive symptoms, remission or problem complexity at Week 16 were not significantly related to CA or PSA at Week 1 (Table 3). Associations between problem number and complexity at Week 1 and the outcomes of interest are provided in Supplementary Section 3.

**Table 3:** Correlations between Week 1 variables and depression outcomes and problem complexity at Weeks 5 and 16 in depressed patients.

	Week 5			Week 16		
	Depression	Remission	PC	Depression	Remission	PC
Week 1	*r_s_* (*p*)	*r_s_* (*p*)	*r_s_* (*p*)	*r_s_* (*p*)	*r_s_* (*p*)	*r_s_* (*p*)
CA	−0.10, *p* = 0.407	−0.18, *p* = 0.157	−0.10, *p* = 0.449	0.09, *p* = 0.532	0.03, *p* = 0.847	−0.07, *p* = 0.641
PSA	−0.27, *p* = 0.026	0.10, *p* = 0.449	−0.16, *p* = 0.206	0.04, *p* = 0.785	0.06, *p* = 0.691	−0.16, *p* = 0.288

CA, causal analysis; PSA, problem-solving analysis; PC, problem complexity; NUM, problem number. Depression scores were adjusted for baseline depression by subtracting Week 1 depression from Week 5 to Week 16 depression. Problem complexity scores were adjusted for baseline problem complexity by subtracting Week 1 problem complexity from Week 5 to Week 16 problem complexity.

### Do Depressed Individuals Have More Complex Problems and Do They Engage in More Analytical Rumination Than Non-depressed Individuals?

#### Between-Group Comparisons of Complex Problems

Depressed patients reported having more problems than non-depressed participants at each week (Week 1: *W* = 407, *p* < 0.001; Week 5: *W* = 357.5, *p* < 0.001; Week 16: *W* = 251, *p* < 0.001). Depressed patients also perceived their problems to be more complex than non-depressed participants at each week (Week 1: *W* = 265, *p* < 0.001; Week 5: *W* = 368, *p* < 0.001; Week 16: *W* = 252.5, *p* < 0.001). Table 2 presents descriptive information for problem number and complexity at each week.

#### Between-Group Comparisons of Analytical Rumination

Depressed patients reported more CA than non-depressed participants at Week 1 (*W* = 951.5, *p* < 0.01) and Week 5 (*W* = 859, *p* < 0.01), but not at Week 16 (*W* = 826.5, *p* = 0.43). The two groups did not differ in PSA at any time (Week 1: *W* = 2065, *p* = 0.64; Week 5: *W* = 1,386, *p* = 0.90; Week 16: *W* = 1070.5, *p* = 0.18). Table 2 presents means and standard deviations for CA and PSA at each week.

### Does Analytical Rumination Decrease Depression and Problem Complexity Over Time?

Based on QQ and influence plots (provided in Supplementary Section 1), we identified four influential observations for each of our multiple regression models. We removed these observations from our analyses, and we report the results excluding influential observations below. Results from models including the influential observations are provided in Supplementary Section 2.

#### Effects of CA and PSA on Depressive Symptoms

Week 5: Table 4 presents the results of the multiple linear regression examining the unique effects of CA and PSA on depressive symptoms, controlling for covariates (baseline depression, problem number, and problem complexity). Baseline depression and problem complexity were significant positive predictors of depression at Week 5. Higher PSA at Week 1 was also uniquely related to less depression at Week 5. No other variables at Week 1 affected depression at Week 5.

**Table 4:** Multiple regression results examining the unique effects of CA and PSA on depressive symptoms at Weeks 5 and 16 (excluding influential observations).

		Depressive symptoms						Remission likelihood						Problem complexity				
		Week 5			Week 16			Week 5		Week 16				Week 5		Week 16		
Week 1 predictors	*β*	SE	*p*	*β*	SE	*p*	*β*	SE	*p*	*β*	SE	*p*	*β*	SE	*p*	*β*	SE	*p*
Depression	0.57	0.22	0.013	0.00	0.37	0.999	−0.26	0.12	0.031	−0.11	0.09	0.227	0.16	0.12	0.189	−0.12	0.18	0.512
Problem number	0.25	0.40	0.535	0.29	0.64	0.724	0.00	0.22	0.988	0.04	0.15	0.793	0.25	0.22	0.251	0.13	0.32	0.692
PCQ	0.71	0.26	0.001	1.24	0.44	0.001	−0.31	0.12	0.001	−0.21	0.11	0.061	0.51	0.15	0.002	0.95	0.21	<001
CA	0.31	0.57	0.590	0.10	0.87	0.908	−0.34	0.28	0.220	0.23	0.23	0.297	0.13	0.30	0.667	0.00	0.42	0.997
PSA	−1.48	0.59	0.016	−0.84	0.90	0.358	0.52	0.30	0.087	0.23	0.21	0.277	−0.60	0.33	0.070	−0.71	0.41	0.093

PCQ, problem complexity questionnaire; CA, causal analysis; PSA, problem solving analysis; β, standardized regression coefficient; SE, standard error.

Week 16: More complex problems at Week 1 predicted more severe symptoms at follow-up. CA, PSA, baseline depression, and problem number did not predict depressive symptoms at follow-up (full results are provided in Table 4).

#### Effects of CA and PSA on Likelihood of Remission

Week 5: Less severe depressive symptoms and less complex problems at Week 1 were uniquely associated with a higher likelihood of remission at Week 5. However, CA, PSA, and problem number at Week 1 were not significant predictors of remission after hospitalization (Table 4).

Week 16: Problem complexity at Week 1 was a negative predictor of remission at Week 16, but this effect was not statistically significant (*p* = 0.061). CA, PSA, baseline depression, and problem number did not predict the likelihood of remission at follow-up (Table 4).

#### Unique Effects of CA and PSA on Problem Complexity

Week 5: In a multiple regression, more complex problems at Week 1 were uniquely associated with more complex problems at Week 5. PSA at Week 1 was a negative predictor of complexity at Week 5, but this effect was not statistically significant (*p* = 0.070). CA, baseline depression, and problem number did not predict problem complexity at Week 5 (Table 4).

Week 16: More complex problems at Week 1 were uniquely associated with more complex problems at Week 16. PSA at Week 1 was a negative predictor of complexity at Week 16, but this effect was not statistically significant (*p* = 0.093). No other variables at Week 1 were unique predictors of problem complexity at Week 16 (Table 4).

#### Results Including Influential Observations

Supplementary Section 2 contains full results for the multiple regression analyses including influential observations (i.e., outliers). In these analyses, problem complexity at baseline did not affect depressive symptoms at Week 5, and the effect of PSA was not statistically significant (*p* = 0.056). Problem complexity had a non-significant effect on depressive symptoms at Week 16 (*p* = 0.063). Its effects on remission from depression and problem complexity at Week 5 were also not statistically significant (*p* = 0.080 and *p* = 0.076). There were no other qualitative differences between findings from analyses with and without the influential observations.

## Discussion

Consistent with our predictions, depressed patients reported having more problems, and they perceived their problems to be more complex than non-depressed participants. They also reported more CA, but differences in CA between the two samples became smaller over the course of treatment. There were no between-groups differences in PSA, meaning that depressed individuals engaged in similar degrees of problem-solving as non-depressed individuals. In correlations and multiple regressions, we found evidence to suggest that depressed patients who engaged in more PSA at the start of their hospitalization were more likely to report decreases in symptoms at the end of their hospitalization. However, engaging in more PSA at the start of hospitalization did not affect depressive symptoms at follow-up. PSA also did not predict the likelihood of remission after hospitalization or at follow-up; however, having less complex problems at the start of hospitalization made it more likely that a patient would remit after hospitalization. Finally, engaging in more CA or PSA at the start of hospitalization did not affect perceived problem complexity after hospitalization or at follow-up. Not surprisingly however, our regression models suggested that more complex problems at baseline were related to more complex problems over time. Overall, our findings suggest that clinically-depressed individuals naturally engage in CA and PSA and that PSA may contribute to a decrease in depressive symptoms over time. However, remission from depression and reductions in perceived problem complexity might only be achieved if problems are initially less complex.

Our findings support the evolutionary perspective in suggesting that clinically depressed patients engage in analytical rumination and that they make cognitive efforts to solve their problems. According to research on coping, rumination forms a part of an unproductive coping mechanism variously referred to as rigid perseveration or submission, and it is considered to be different from problem-solving (i.e., strategizing, planning, or instrumental action; Skinner et al., 2003). In various studies, depression has been associated with deficits in cognitive domains such as attention, working memory, and different forms of learning, as assessed by neuropsychological tests (Lee et al., 2012; Rock et al., 2014). These deficits have been interpreted as evidence to suggest that depressed individuals have difficulties engaging in productive thinking or problem-solving (Lyubomirsky and Nolen-Hoeksema, 1995; Nolen-Hoeksema et al., 2008). Across different research domains, rumination and depressive thinking have often been categorized as being unconstructive, leading to negative outcomes for the self or stressful situations.

In contrast to this view, we found evidence that depressed patients ruminate (i.e., think repetitively and persistently) about ways to address their problems, which may play a role in decreasing their symptoms over time. We observed the same degree of strategizing, goal-oriented thinking, and learning from mistakes in inpatients being hospitalized for MDD as we did in healthy controls, suggesting that even individuals with severe depression may engage in effective problem-solving. According to coping researchers, problem-solving is different from rumination because problem-solving coordinates actions in the environment (Skinner et al., 2003). We suggest that problem-solving in MDD might also involve an inner analysis of preferences and available options for ways to deal with particularly complex circumstances. Moreover, we suggest that this mental activity is demanding and consumes cognitive resources. Decrements in neuropsychological assessment of cognition observed in MDD might therefore emerge due to rumination (Andrews and Thomson, 2009). Accordingly, studies that do not provide depressed individuals the opportunity to ruminate (e.g., by ensuring attention is well-controlled by the demands of the laboratory task) do not find these cognitive deficits (Gotlib and Joormann, 2010).

Our findings should be considered in the context of other research suggesting that rumination (as assessed with the Ruminative Response Styles Questionnaire) is associated with benefits for cognitive performance, such as verbal comprehension (Penney et al., 2015). When controlling for depressive symptoms, a purposeful turning inward to engage in cognitive problem-solving (i.e., reflective pondering) has been positively associated with performance on intelligence tests. However, engaging in passive comparisons with unachieved standards (i.e., brooding) was not associated with this performance (du Pont et al., 2020). Although associations between analytical rumination and cognitive performance have not been examined, it is possible that individuals with better verbal or cognitive abilities may be more likely to engage in CA or PSA. These abilities might also facilitate components of analytical rumination. The relationship between depressive rumination and cognitive ability appears to be complex, and future work might examine whether cognitive performance in various domains is a moderator of CA and PSA.

Importantly, when combined with previous studies of the ARH (Bartoskova et al., 2018; Maslej et al., 2019), the finding that PSA may be related to a decrease in depressive symptoms over time helps elucidate the temporal process of analytical rumination. Previous work has documented a link between sadness and CA (Maslej et al., 2019) and a short-term effect of PSA on decreases in sadness and symptoms of depression. However, it was not clear whether this problem-solving in depression is adaptive, i.e., whether it is associated with future relief of depressive symptoms or remission. According to cognitive models of depression, depressed people are biased in how they process information, leading them to perceive negative events as hopeless or outside of their control (Beck, 1976). When people with this bias experience a negative event and become sad, their ruminations are thought to prevent them from actively coping with their issues (Lyubomirsky and Nolen-Hoeksema, 1995). According to this view, it was possible that ruminative thoughts might have prevented individuals from engaging in effective problem-solving, exacerbating their depression. Our finding of this negative longitudinal link between PSA and depression in a clinically depressed sample implies that the clinical views or cognitive theories may not be entirely correct. Instead, it suggests that effective problem-solving can occur in the presence of clinical depression, possibly forming part of a functional process that decreases depressive symptoms.

Although we did not find evidence of a direct effect of CA on depression outcomes, CA is hypothesized to affect symptoms indirectly, i.e., *via* PSA. Contrary to our predictions, however, we did not find evidence that PSA led to the remission from depression, at least in the time frames that we measured (i.e., after hospitalization or at a 16-week follow up). Because baseline problem complexity was a negative predictor of remission after hospitalization, it is possible that the utility of PSA depends on initial problem complexity. For individuals experiencing very complex problems at the outset, PSA may not contribute to remission. Another possibility is that our analyses involving remission were underpowered, given that there were few remitters coupled with a small sample size, particularly at follow-up. Grouping individuals into remitters or non-remitters may have also obscured meaningful variations in symptoms. In support of this explanation, PSA was a non-significant predictor of remission after hospitalization. Nevertheless, although PSA decreases depressive symptoms over time, it may not impact remission.

In contrast to our expectation, we did not find evidence that CA or PSA contributed to problem complexity over time. Although engaging in analytical rumination is hypothesized to help individuals address their problems (Andrews and Thomson, 2009), it may not actually lead individuals to perceive their problems as being any less complex. Future research might involve developing a measure of the degree to which individuals have made progress at addressing their problems, to better elucidate the mechanisms by which PSA exerts its effects on symptoms. Solving complex problems may benefit them from developing a good understanding of their causes, so that problem-solving efforts can be directed toward the source of the issues (Barbic et al., 2014). Thus, future research might determine whether the presence of CA impacts the longitudinal effect of PSA on depression.

### Limitations

Our results must be interpreted in the context of several methodological limitations. First, although the finding that analytical rumination may play a role in decreasing depressive symptoms is promising, the actual mechanism of healing remains unknown. It is possible that simply making progress at thinking through the problem, without actually taking steps to address it in real life, alleviates depressive symptoms. However, to achieve remission or a large decrease in symptoms, it may be necessary to address the problem and alleviate the negative circumstance that initially triggered the depressive episode. This may explain why PSA seemed to decrease symptoms, but it did not contribute to remission. Future research might examine whether PSA promotes actions or efforts to actually solve problems in real life, as well as the impact of these actions or efforts on depressive symptoms.

Furthermore, participants in our study retrospectively reported how often they had been ruminating over the past 2 weeks. To comprehensively capture changes in rumination over depression, and to determine their impact on changes in depressive symptoms more precisely, study designs involving much more frequent assessment are required, such as experience sampling methods (ESM; Hektner et al., 2007). It is also important to note that we reported findings from our regression models excluding influential observations, which appeared to be obscuring our effects. With influential observations included, some of our findings are no longer statistically significant, most notably the effect of PSA on depressive symptoms after hospitalization (Supplementary Section 2). We determined our criteria for identifying influential observations *a priori*, basing our decision on the diagnostic plots provided in Supplementary Section 1. However, to determine the presence and influence of these observations, we analyzed the data both with and without them. Given that these analyses yielded slightly different findings, the longitudinal effect of PSA on depression in individuals with MDD should be replicated.

Our study adds value to the literature by testing ARH predictions in a clinically depressed sample. We specifically focused on hospitalized inpatients to evaluate the claim that severe depression prevents individuals from engaging in effective problem-solving (Lyubomirsky and Nolen-Hoeksema, 1995). At the same time, using a severely depressed sample introduced confounds (i.e., related to hospitalization). All of these patients were receiving antidepressant and/or benzodiazepine drugs at the time of the study. These patients also had access to psychosocial interventions (such as group therapy, art therapy or ergotherapy), which may have impacted their ability to ruminate or solve problems. For example, being hospitalized might have prevented inpatients from taking any real actions to solve their problems. Alternatively, the psychosocial interventions may have facilitated efforts to problem solve, for example, by teaching patients how to wait through or cope with adversity, how to elicit attention and support from others, or even how to understand their problems more deeply. In one study of hospitalized inpatients, 85% of depressed inpatients perceived hospitalization as providing protection from the external world and 75% perceived it as an opportunity to recover their own existence (Biancosino et al., 2009). It is therefore possible that our findings would not generalize to untreated individuals. On the other hand, the ARH predicts that theoretical claims might be less plausible for people taking antidepressants, because these medications might lead to alterations in the depressive symptoms hypothesized to be adaptive, including PSA. In addition, we investigated the role of PSA at the start of hospitalization, which would not have been affected by interventions related to hospitalization. Nevertheless, an important future direction for this work involves replicating the study with depressed individuals who are not taking antidepressants or receiving psychosocial interventions.

Another limitation is related to our inclusion criteria. Only patients with a main diagnosis of MDD were included in our study. However, in clinical practice, depressive disorders are commonly comorbid, i.e., with anxiety symptoms and anxiety disorders. It has been claimed that the rigid application of a diagnostic criteria does not adequately identify differences between patients (Wu and Fang, 2014), which seems to be a key factor in determining the trait’s evolved function. Thus, although the inclusion and exclusion criteria for our MDD sample were strict, our results might not apply to all depressed individuals.

### Research Implications

These findings have at least three practical research implications. First, they provide insight into the course of depression mainly in terms of individual transitions from MDD to health during treatment. Several factors, both static and dynamic, have been previously described as having a major role in remission from depression (i.e., psychiatric comorbidities, suicidal tendencies, prior treatment for depression, and obesity; Saragoussi et al., 2017; Vitriol et al. 2018). According to the ARH, one of these factors is the analytical rumination preceding the remission from depression. Previous work on the ARH suggests that the process of analytical rumination (i.e., CA followed by PSA) decreases depressive symptoms in the short-term (Bartoskova et al., 2018). Our study extends on this work by examining the role of analytical rumination on symptoms of depression in the long-term, with some evidence that engaging in PSA at the start of treatment is associated with decreased symptoms following treatment. In other words, clinically depressed individuals who were ruminating about ways to address their problems or to avoid similar problems in the future were more likely to report decreases in symptoms over time. Thus, PSA may play a role in the transition from MDD to health; however, the mechanism by which PSA reduces symptoms remains a direction for future work. Our findings suggest that efforts to elucidate the cause of problems or find ways to solve them may not result in perceptions that problems are any less complex or difficult. However, progress toward solving the problems, even if they remain complex, may be what alleviates depressive symptoms. Instead, problem complexity appears to be a predictive factor; achieving remission from depression may depend on the initial complexity of problems. If problems are particularly complex, recovery may require more time or intervention. However, this former possibility is inconsistent with the finding that PSA did not affect depressive symptoms or the likelihood of remission after 16 weeks. Nevertheless, given the recurrent nature of MDD, it is important to investigate the factors contributing to improvement in mental health, as well as those that contribute to its decline.

Second, our findings might inform our understanding of the high prevalence and recurrence of MDD. Estimates show that depression affects 4.4% of the global population (over 300 million people) at any given time (World Health Organization, 2017), with most of them experiencing depressive relapse within a few years (Hardeveld et al., 2010). By providing evidence that depression is associated with more problems that are perceived to be more complex, we suggest that the reason why depression is so prevalent and recurrent is because modern humans face life changing events, such as economic inequality, migration, globalization, environmental pollution, etc. These events or issues are complex, they do not resolve easily, and they require prolonged analysis. This claim is supported by evidence that depression is more common in developed societies (Hidaka, 2012; Liu et al., 2019). Nonetheless, future research should focus on examining whether ruminating analytically on such intricate situations helps one to cope with them.

Third, our results shed some light on the etiology and treatment of depression. According to evolutionary theory, several etiological pathways exist to each of the subtypes of depression. These paths differ with respect to the ideal treatment strategy. Specifically, the ARH suggests that the treatment of depressive episodes associated with high levels of analytical rumination is productive when it helps depressed individuals identify and solve important problems in their lives (Andrews and Thomson, 2009). Problem-solving therapy (PST) may be the best example since it aims to facilitate adaptive problem-solving attitudes and skills (Haley, 1987). Although PST was shown to be effective in the treatment of depression (Bell and D᾽Zurilla, 2009; Cuijpers et al., 2018), further research investigating the suitability of such therapy for the patients who ruminate would be pertinent.

## Data Availability Statement

Our data are openly available online at: https://mfr.ca-1.osf.io/render?url=https://osf.io/7f2jn/?direct%26mode=render%26action=download%26mode=render.

## Ethics Statement

The studies involving human participants were reviewed and approved by the procedure was approved by the National Institute of Mental Health (NIMH) Ethics Board in Klecany (approval number 49/16). The patients/participants provided their written informed consent to participate in this study.

## Author Contributions

MS, MB, MPa, GV, and MPr designed the study. MS, MPa, GV, and MB participated in data collection and in management of the assessments. MM and JS analyzed the data. MS, MM, and JS drafted the manuscript and discussed it with PA and MPr. All authors provided the comments to the manuscript.

## Conflict of Interest

The authors declare that the research was conducted in the absence of any commercial or financial relationships that could be construed as a potential conflict of interest.

## Supplementary Material

The Supplementary Material for this article can be found online at: https://www.frontiersin.org/articles/10.3389/fpsyg.2020.01344/full#supplementary-material.

## References

[ref1] Abramson L. Y. Metalsky G. I. Alloy L. B. (1989). Hopelessness depression: a theory-based subtype of depression. Psychol. Rev. 96, 358–372. 10.1037/0033-295X.96.2.358

[ref2] American Psychiatric Association (2013). Diagnostic and statistical manual of mental disorders. 5th Edn. Arlington, VA: American Psychiatric Association.

[ref3] Andrews P. W. Thomson J. A. (2009). The bright side of being blue: depression as an adaptation for analyzing complex problems. Psychol. Rev. 116, 620–654. 10.1037/a0016242, PMID: 19618990 PMC2734449

[ref4] Barbic S. P. Durisko Z. Andrews P. W. (2014). Measuring the bright side of being blue: a new tool for assessing analytical rumination in depression. PLoS One 9:e112077. 10.1371/journal.pone.0112077, PMID: 25397902 PMC4232398

[ref5] Bartoskova M. Sevcikova M. Durisko Z. Maslej M. M. Barbic S. P. Preiss M. (2018). The form and function of depressive rumination. Evol. Hum. Behav. 39, 277–289. 10.1016/j.evolhumbehav.2018.01.005

[ref6] Beck A. T. (1976). Cognitive therapy and the emotional disorders. New York: Int. Univ Press.

[ref7] Bell A. C. D᾽Zurilla T. J. (2009). Problem-solving therapy for depression: a meta-analysis. Clin. Psychol. Rev. 29, 348–353. 10.1016/j.cpr.2009.02.003, PMID: 19299058

[ref8] Biancosino B. Marmai L. Borsari B. Padovani S. Magri V. Bertasi R.. (2009). Patient opinions on the experiences of psychiatric hospitalization: a qualitative study. Riv. Psichiatr. 44, 122–133. PMID: 20066814

[ref9] Cook R. D. (1977). Detection of influential observation in linear regression. Technometrics 19, 15–18. 10.2307/1268249

[ref10] Cousineau D. (2005). Confidence intervals in within-subject designs: a simpler solution to Loftus and Masson’s method. Tutor. Quant. Methods Psychol. 1, 42–45. 10.20982/tqmp.01.1.p042

[ref11] Cristobal E. Flavian C. Guinaliu M. (2007). Perceived e-service quality (PeSQ) measurement validation and effects on consumer satisfaction and web site loyalty. J. Serv. Theory Pract. 17, 317–340. 10.1108/09604520710744326

[ref12] Cuijpers P. de Wit L. Kleiboer A. Karyotaki E. Ebert D. D. (2018). Problem-solving therapy for adult depression: an updated meta-analysis. Eur. Psychiatry 48, 27–37. 10.1016/j.eurpsy.2017.11.006, PMID: 29331596

[ref13] du Pont A. Karbin Z. Rhee S. H. Corley R. P. Hewitt J. K. Friedman N. P. (2020). Differential associations between rumination and intelligence subtypes. Intelligence 78:101420. 10.1016/j.intell.2019.101420 PMC7453434 32863476

[ref14] Durisko Z. Mulsant B. H. Andrews P. W. (2015). An adaptationist perspective on the etiology of depression. J. Affect. Disord. 172, 315–323. 10.1016/j.jad.2014.09.032, PMID: 25451432

[ref15] Durisko Z. Mulsant B. H. McKenzie K. Andrews P. W. (2016). Using evolutionary theory to guide mental health research. Can. J. Psychiatr. 61, 159–165. 10.1177/0706743716632517, PMID: 27254091 PMC4813423

[ref16] Fleagle J. G. (2013). “Adaptation, evolution, and systematics” in Primate adaptation and evolution. 3rd Edn. Academic press. 1–7.

[ref17] Fox J. Friendly G. G. Graves S. Heiberger R. Monette G. Nilsson H. (2007). The car package. R Foundation for Statistical Computing.

[ref18] Friedrich M. J. (2017). Depression is the leading cause of disability around the world. JAMA 317, 1517–1517. 10.1001/jama.2017.3828, PMID: 28418490

[ref19] George D. Mallery P. (2003). SPSS for windows step by step: A simple guide and reference. 11.0 update. 4th Edn. Boston: Allyn & Bacon.

[ref20] Gotlib I. H. Joormann J. (2010). Cognition and depression: current status and future directions. Annu. Rev. Clin. Psychol. 6, 285–312. 10.1146/annurev.clinpsy.121208.131305, PMID: 20192795 PMC2845726

[ref21] Haaga D. A. Dyck M. J. Ernst D. (1991). Empirical status of cognitive theory of depression. Psychol. Bull. 110, 215–236. 10.1037/0033-2909.110.2.215, PMID: 1946867

[ref22] Hagen E. H. (2011). Evolutionary theories of depression: a critical review. Can. J. Psychiatr. 56, 716–726. 10.1177/070674371105601203, PMID: 22152640

[ref23] Haley J. (1987). Problem-solving therapy. San Francisco, CA: Jossey-Bass.

[ref24] Hardeveld F. Spijker J. De Graaf R. Nolen W. A. Beekman A. T. F. (2010). Prevalence and predictors of recurrence of major depressive disorder in the adult population. Acta Psychiatr. Scand. 122, 184–191. 10.1111/j.1600-0447.2009.01519.x, PMID: 20003092

[ref25] Hektner J. M. Schmidt J. A. Csikszentmihalyi M. (2007). Experience sampling method: Measuring the quality of everyday life. London: Sage.

[ref26] Hermens M. L. Adèr H. J. van Hout H. P. Terluin B. van Dyck R. de Haan M. (2006). Administering the MADRS by telephone or face-to-face: a validity study. Ann. General Psychiatry 5:3. 10.1186/1744-859X-5-3, PMID: 16553958 PMC1435896

[ref27] Hidaka B. H. (2012). Depression as a disease of modernity: explanations for increasing prevalence. J. Affect. Disord. 140, 205–214. 10.1016/j.jad.2011.12.036, PMID: 22244375 PMC3330161

[ref28] Hothorn T. Hornik K. van de Wiel M. A. Zeileis A. (2008). Implementing a class of permutation tests: the coin package. J. Stat. Softw. 28, 1–23. 10.18637/jss.v028.i0827774042

[ref29] Keller M. C. Neale M. C. Kendler K. S. (2007). Association of different adverse life events with distinct patterns of depressive symptoms. Am. J. Psychiatry 164, 1521–1529. 10.1176/appi.ajp.2007.06091564, PMID: 17898343

[ref30] Keller M. C. Nesse R. M. (2006). The evolutionary significance of depressive symptoms: different adverse situations lead to different depressive symptom patterns. J. Pers. Soc. Psychol. 91, 316–330. 10.1037/0022-3514.91.2.316, PMID: 16881767

[ref31] Krogsbøll L. T. Hróbjartsson A. Gøtzsche P. C. (2009). Spontaneous improvement in randomised clinical trials: meta-analysis of three-armed trials comparing no treatment, placebo and active intervention. BMC Med. Res. Methodol. 9:1. 10.1186/1471-2288-9-1 19123933 PMC2628943

[ref32] Lee R. S. Hermens D. F. Porter M. A. Redoblado-Hodge M. A. (2012). A meta-analysis of cognitive deficits in first-episode major depressive disorder. J. Affect. Disord. 140, 113–124. 10.1016/j.jad.2011.10.023, PMID: 22088608

[ref33] Levenson R. W. (1999). The intrapersonal functions of emotion. Cognit. Emot. 13, 481–504. 10.1080/026999399379159

[ref34] Lewinsohn P. M. Joiner T. E. Jr. Rohde P. (2001). Evaluation of cognitive diathesis-stress models in predicting major depressive disorder in adolescents. J. Abnorm. Psychol. 110, 203–215. 10.1037/0021-843X.110.2.203, PMID: 11368074

[ref35] Liu Q. He H. Yang J. Feng X. Zhao F. Lyu J. (2019). Changes in the global burden of depression from 1990 to 2017: findings from the Global Burden of Disease study. J. Psychiatr. Res. 126, 134–140. 10.1016/j.jpsychires.2019.08.002, PMID: 31439359

[ref36] Lyubomirsky S. Nolen-Hoeksema S. (1993). Self-perpetuating properties of dysphoric rumination. J. Pers. Soc. Psychol. 65, 339–349. 10.1037/0022-3514.65.2.339, PMID: 8366423

[ref37] Lyubomirsky S. Nolen-Hoeksema S. (1995). Effects of self-focused rumination on negative thinking and interpersonal problem solving. J. Pers. Soc. Psychol. 69, 176–190. 10.1037/0022-3514.69.1.176, PMID: 7643299

[ref38] Maslej M. Rheaume A. R. Schmidt L. A. Andrews P. W. (2019). Using expressive writing to test an evolutionary hypothesis about depressive rumination: sadness coincides with causal analysis of a personal problem, not problem-solving analysis. Evol. Psychol. Sci. 6, 1–17. 10.1007/s40806-019-00219-8

[ref39] Moberly N. J. Watkins E. R. (2008). Ruminative self-focus, negative life events, and negative affect. Behav. Res. Therapy 46, 1034–1039. 10.1016/j.brat.2008.06.004, PMID: 18684437 PMC2682175

[ref40] Montgomery S. A. Åsberg M. (1979). A new depression scale designed to be sensitive to change. Br. J. Psychiatry 134, 382–389. 10.1192/bjp.134.4.382, PMID: 444788

[ref41] Nesse R. M. (1990). Evolutionary explanations of emotions. Hum. Nat. 1, 261–289. 10.1007/BF02733986, PMID: 24222085

[ref42] Nesse R. M. (2000). Is depression an adaptation? Arch. Gen. Psychiatry 57, 14–20. 10.1001/archpsyc.57.1.14, PMID: 10632228

[ref43] Nettle D. (2004). Evolutionary origins of depression: a review and reformulation. J. Affect. Disord. 81, 91–102. 10.1016/j.jad.2003.08.009, PMID: 15306134

[ref44] Nolen-Hoeksema S. (1991). Responses to depression and their effects on the duration of depressive episodes. J. Abnorm. Psychol. 100, 569–582. 10.1037/0021-843X.100.4.569, PMID: 1757671

[ref45] Nolen-Hoeksema S. Wisco B. E. Lyubomirsky S. (2008). Rethinking rumination. Perspect. Psychol. Sci. 3, 400–424. 10.1111/j.1745-6924.2008.00088.x, PMID: 26158958

[ref600] Open Science Framework (2020a). Testing the ARH_Data. Available at: https://mfr.ca-1.osf.io/render?url=https://osf.io/7f2jn/?direct%26mode=render%26action=download%26mode=render (Accessed April 20, 2020).

[ref700] Open Science Framework (2020b). Testing the ARH_Study code. Available at: https://mfr.ca-1.osf.io/render?url=https://osf.io/jsv2k/?direct%26mode=render%26action=download%26mode=render (Accessed May 9, 2020).

[ref48] Penney A. M. Miedema V. C. Mazmanian D. (2015). Intelligence and emotional disorders: is the worrying and ruminating mind a more intelligent mind? Pers. Individ. Differ. 74, 90–93. 10.1016/j.paid.2014.10.005

[ref49] Posternak M. A. Miller I. (2001). Untreated short-term course of major depression: a meta-analysis of outcomes from studies using wait-list control groups. J. Affect. Disord. 66, 139–146. 10.1016/S0165-0327(00)00304-9, PMID: 11578666

[ref50] Ripley B. Venables B. Bates D. M. Hornik K. Gebhardt A. Firth D. (2013). Package ‘mass’. Cran R 538.

[ref51] Rock P. L. Roiser J. P. Riedel W. J. Blackwell A. D. (2014). Cognitive impairment in depression: a systematic review and meta-analysis. Psychol. Med. 44, 2029–2040. 10.1017/S0033291713002535, PMID: 24168753

[ref52] Saragoussi D. Touya M. Haro J. M. Jönsson B. Knapp M. Botrel B.. (2017). Factors associated with failure to achieve remission and with relapse after remission in patients with major depressive disorder in the PERFORM study. Neuropsychiatr. Dis. Treat. 13, 2151–2165. 10.2147/NDT.S136343, PMID: 28860772 PMC5558880

[ref53] Sheehan D. V. Lecrubier Y. Sheehan K. H. Janavs J. Weiller E. Keskiner A. (1997). The MINI International Neuropsychiatric Interview (MINI). A short diagnostic structured interview: reliability and validity according to the CIDI. Eur. Psychiatry 12, 232–241. 10.1016/S0924-9338(97)83297-X

[ref54] Skinner E. A. Edge K. Altman J. Sherwood H. (2003). Searching for the structure of coping: a review and critique of category systems for classifying ways of coping. Psychol. Bull. 129, 216–219. 10.1037/0033-2909.129.2.216, PMID: 12696840

[ref300] Treynor W. Gonzalez R. Nolen-Hoeksema S. (2003). Rumination reconsidered: a psychometric analysis. Cognit. Ther. Res. 27, 247–259. 10.1023/A:102391031556124712535

[ref55] Vitriol V. Cancino A. Serrano C. Ballesteros S. Potthoff S. (2018). Remission in depression and associated factors at different assessment times in primary care in Chile. Clin. Prac. Epidemiol. Ment. Health 14, 78–88. 10.2174/1745017901814010078, PMID: 29643931 PMC5876920

[ref56] Whiteford H. A. Harris M. G. McKeon G. Baxter A. Pennell C. Barendregt J. J.. (2013). Estimating remission from untreated major depression: a systematic review and meta-analysis. Psychol. Med. 43, 1569–1585. 10.1017/S0033291712001717, PMID: 22883473

[ref57] World Health Organization (2017). Depression and other common mental disorders: global health estimates (No. WHO/MSD/MER/2017.2). World Health Organization.

[ref58] Wu Z. Fang Y. (2014). Comorbidity of depressive and anxiety disorders: challenges in diagnosis and assessment. Shanghai Arch. Psychiatry 26, 227–231. 10.3969/j.issn.1002-0829.2014.04.006, PMID: 25317009 PMC4194005

[ref59] Zimmerman M. Posternak M. A. Chelminski I. (2004). Derivation of a definition of remission on the Montgomery–Asberg depression rating scale corresponding to the definition of remission on the Hamilton rating scale for depression. J. Psychiatr. Res. 38, 577–582. 10.1016/j.jpsychires.2004.03.007, PMID: 15458853

