# Supplementary material for: Testing the Analytical Rumination Hypothesis: Exploring the Longitudinal Effects of Problem Solving Analysis on Depression
Source: Front Psychol. 2020 Jul 2;11:1344. doi: 10.3389/fpsyg.2020.01344 (PMC7344354; doi:10.3389/fpsyg.2020.01344)
Supplement: Supplementary file 1 [file Data_Sheet_1.DOCX]

Table 4

*Multiple regression results examining the unique effects of CA and PSA on depressive symptoms at Weeks 5 and 16 (excluding influential observations)*

|  | | | Depressive symptoms | | | | | | | | Remission likelihood | | | | | | | Problem complexity | | | | | | | |
| --- | --- | --- | --- | --- | --- | --- | --- | --- | --- | --- | --- | --- | --- | --- | --- | --- | --- | --- | --- | --- | --- | --- | --- | --- | --- |
|  | | | Week 5 | | | | Week 16 | | | | Week 5 | | | Week 16 | | | | | Week 5 | | | | Week 16 | | |
| Week 1 predictors | β | SE | | *p* | β | SE | | *p* | β | SE | | *p* | β | | SE | *p* | β | | | SE | *p* | β | | SE | *p* |
| Depression | 0.57 | 0.22 | | .013 | 0.00 | 0.37 | | .999 | -0.26 | 0.12 | | .031 | -0.11 | | 0.09 | .227 | 0.16 | | | 0.12 | .189 | -0.12 | | 0.18 | .512 |
| Problem number | 0.25 | 0.40 | | .535 | 0.29 | 0.64 | | .724 | 0.00 | 0.22 | | .988 | 0.04 | | 0.15 | .793 | 0.25 | | | 0.22 | .251 | 0.13 | | 0.32 | .692 |
| PCQ | 0.71 | 0.26 | | .001 | 1.24 | 0.44 | | .001 | -0.31 | 0.12 | | .001 | -0.21 | | 0.11 | .061 | 0.51 | | | 0.15 | .002 | 0.95 | | 0.21 | <.001 |
| CA | 0.31 | 0.57 | | .590 | 0.10 | 0.87 | | .908 | -0.34 | 0.28 | | .220 | 0.23 | | 0.23 | .297 | 0.13 | | | 0.30 | .667 | 0.00 | | 0.42 | .997 |
| PSA | -1.48 | 0.59 | | .016 | -0.84 | 0.90 | | .358 | 0.52 | 0.30 | | .087 | 0.23 | | 0.21 | .277 | -0.60 | | | 0.33 | .070 | -0.71 | | 0.41 | .093 |

*Note.* PCQ = Problem complexity questionnaire; CA = Causal analysis; PSA = Problem solving analysis; β = standardized regression coefficient; SE = Standard error.

***Supplementary Section 1***
 *QQ Plot: Effects of Week 1 CA, PSA and covariates on Week 5 depressive symptoms*


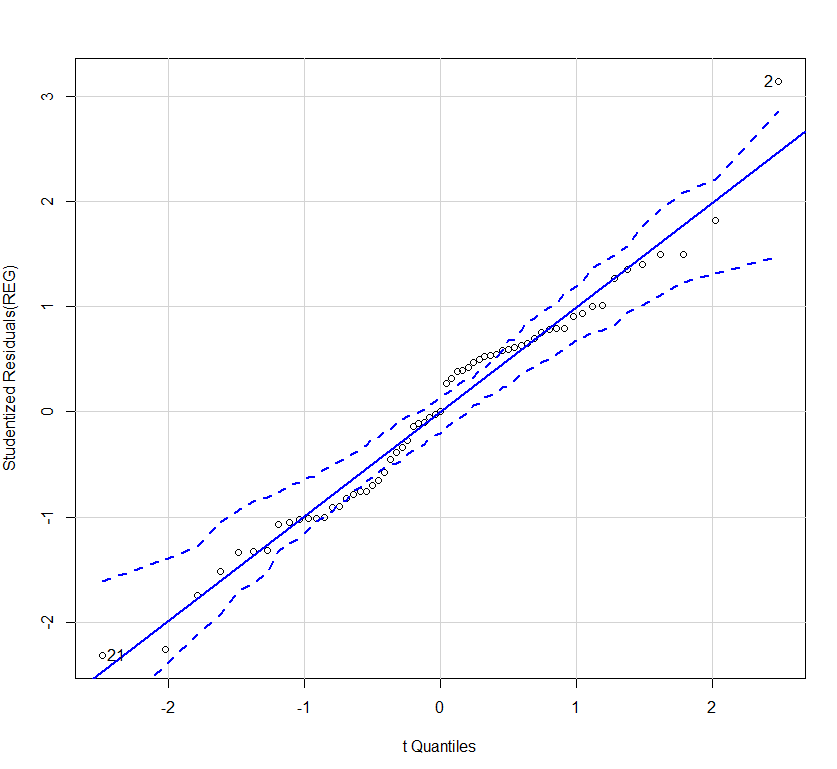
 *Influence Plot: Effects of Week 1 CA, PSA and covariates on Week 5 depressive symptoms*


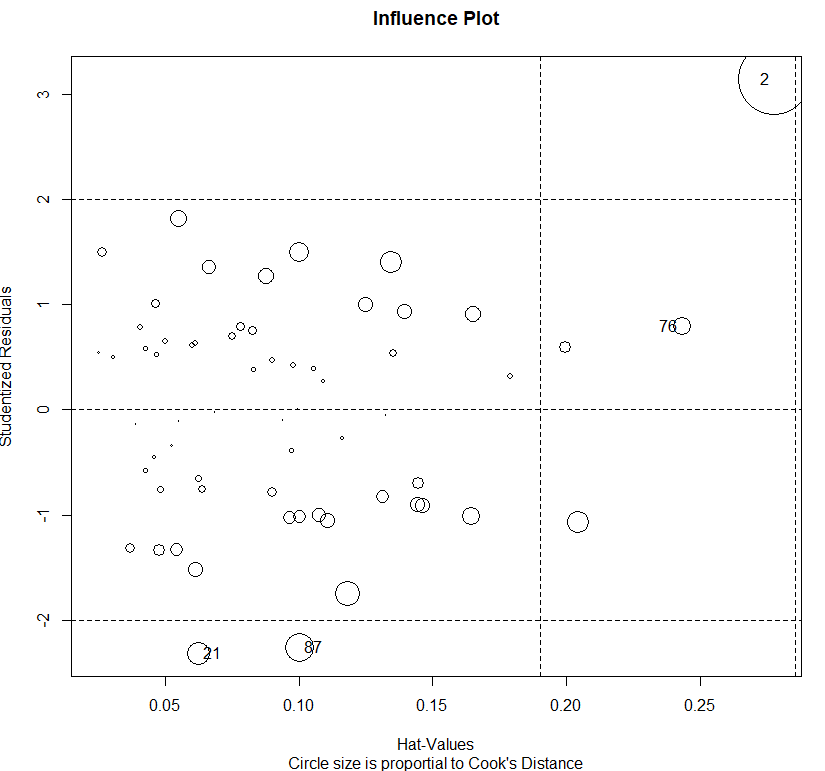


*QQ Plot: Effects of Week 1 CA, PSA and covariates on Week 16 depressive symptoms* ***
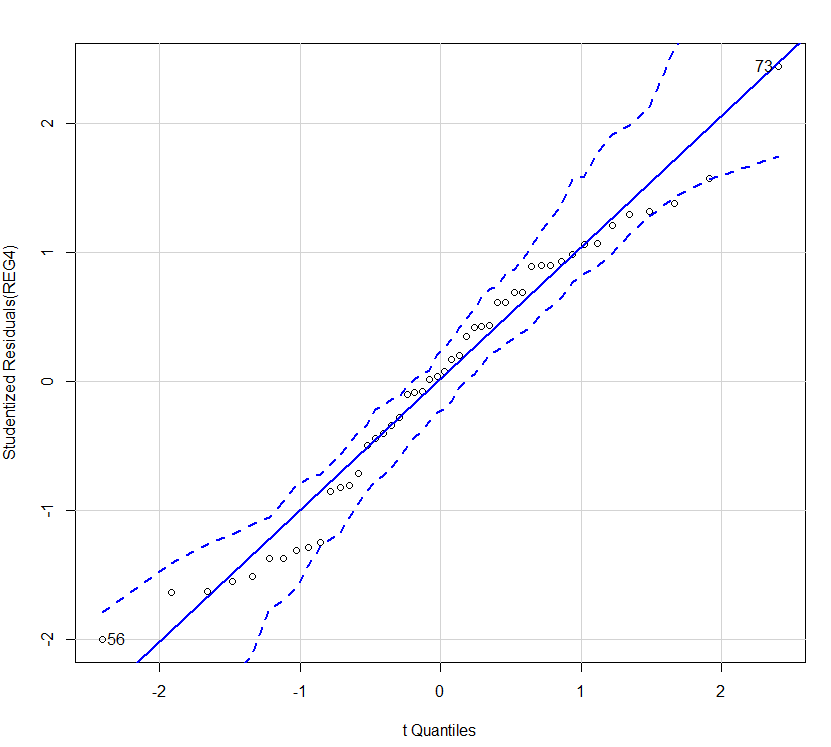
***

*Influence Plot: Effects of Week 1 CA, PSA and covariates on Week 16 depressive symptoms*


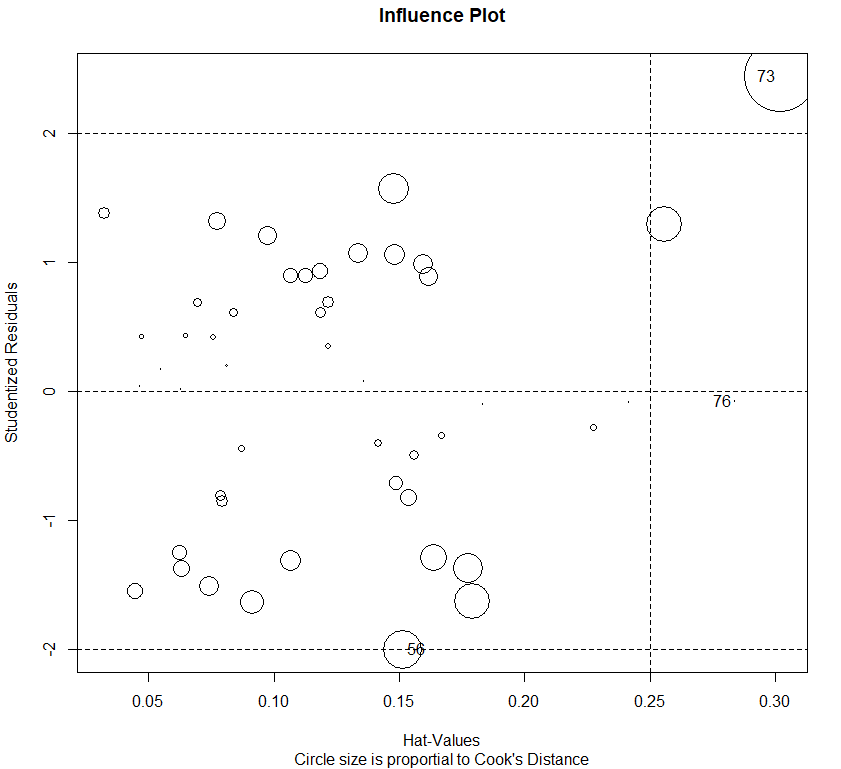


*QQ Plot: Effects of Week 1 CA, PSA and covariates on Week 5 perceived problem complexity*
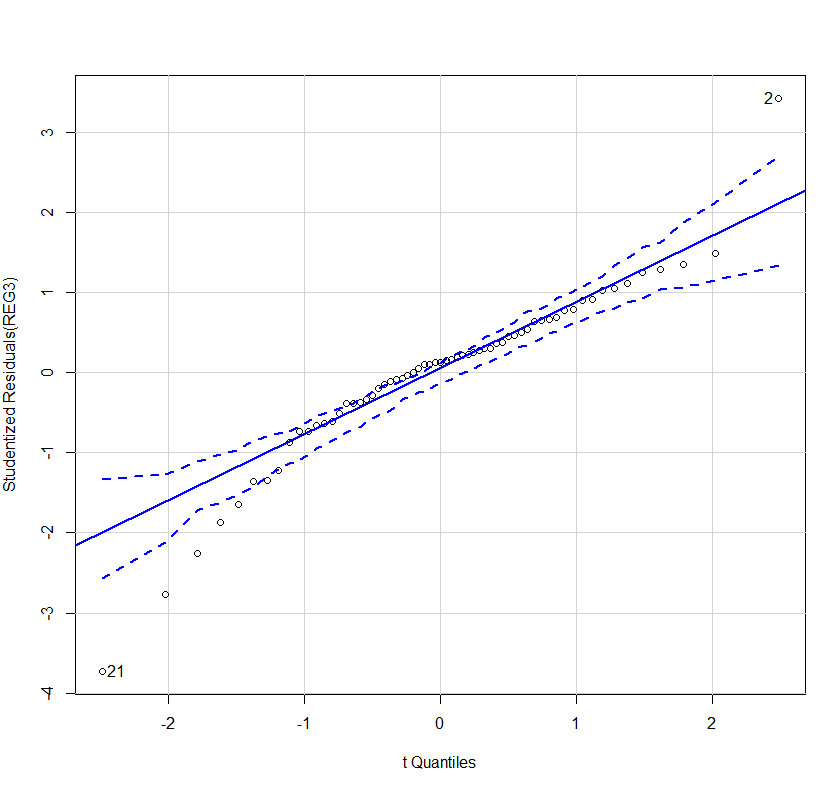


*Influence Plot: Effects of Week 1 CA, PSA and covariates on Week 5 perceived problem complexity

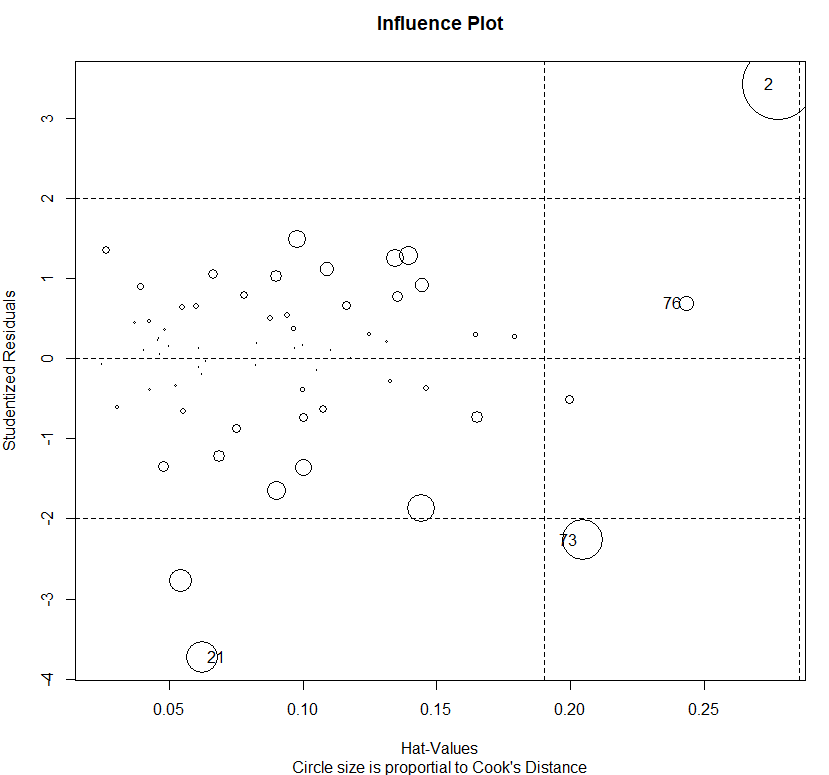
*

*QQ Plot: Effects of Week 1 CA, PSA and covariates on Week 16 perceived problem complexity

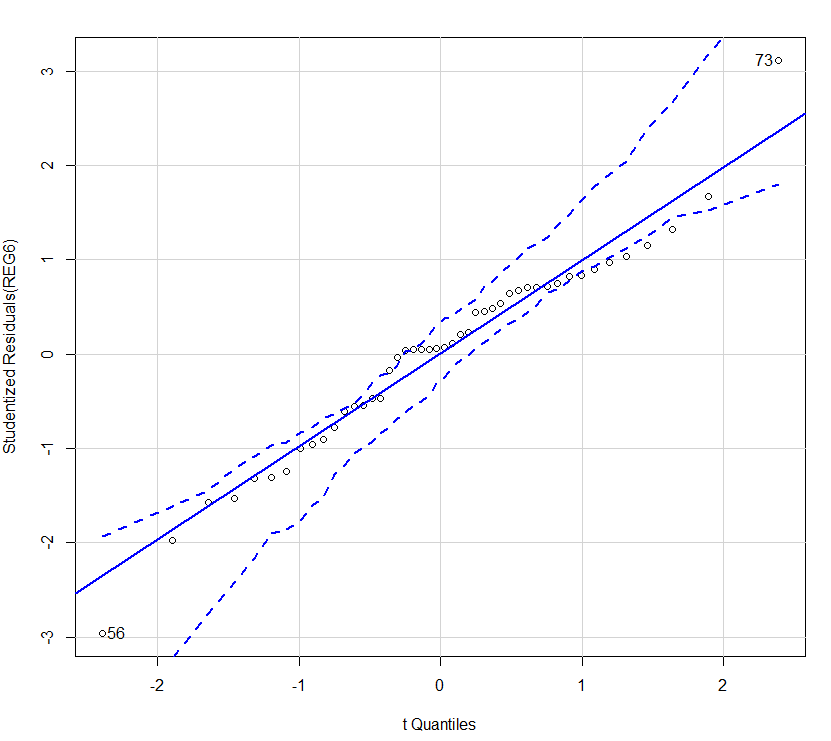

Influence Plot: Effects of Week 1 CA, PSA and covariates on Week 16 perceived problem complexity*


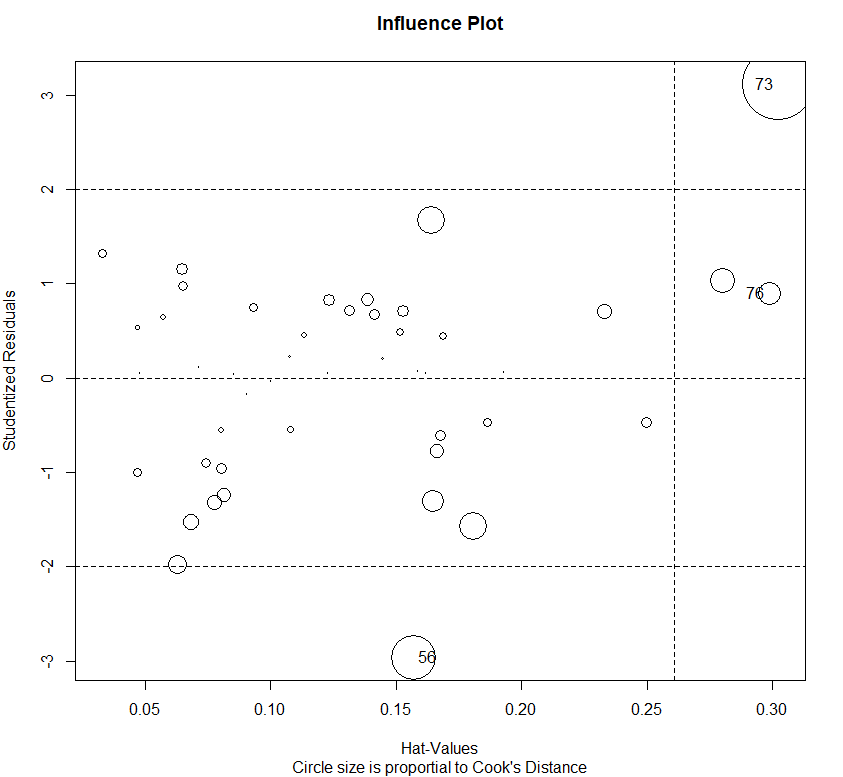


Supplementary Section 2

*Multiple regression results examining the unique effects of CA and PSA on depressive symptoms at Weeks 5 and 16 (including influential observations)*

|  | | | Depressive symptoms | | | | | | | | Remission likelihood | | | | | | | Problem complexity | | | | | | |
| --- | --- | --- | --- | --- | --- | --- | --- | --- | --- | --- | --- | --- | --- | --- | --- | --- | --- | --- | --- | --- | --- | --- | --- | --- |
|  | | | Week 5 | | | | Week 16 | | | | Week 5 | | | Week 16 | | | | Week 5 | | | | Week 16 | | |
| Week 1 predictors | β | SE | | *p* | β | SE | | *p* | β | SE | | *p* | β | | SE | *p* | β | | SE | *p* | β | | SE | *p* |
| Depression | 0.53 | 0.25 | | .039 | -0.04 | 0.36 | | .921 | -0.15 | 0.08 | | .061 | -0.05 | | 0.07 | .487 | 0.13 | | 0.15 | .366 | -0.23 | | 0.20 | .264 |
| Problem number | 0.46 | 0.46 | | 0.324 | 0.31 | 0.65 | | .643 | -0.06 | 0.16 | | .690 | -0.03 | | 0.13 | .820 | 0.27 | | 0.27 | .330 | 0.30 | | 0.37 | .416 |
| PCQ | 0.18 | 0.23 | | 0.486 | 0.76 | 0.40 | | .063 | -0.13 | 0.07 | | .080 | -0.12 | | 0.08 | .145 | 0.27 | | 0.15 | .076 | 0.61 | | 0.23 | .010 |
| CA | 0.55 | 0.65 | | .401 | 0.25 | 0.94 | | .796 | -0.30 | 0.21 | | .150 | 0.13 | | 0.19 | .497 | 0.45 | | 0.38 | .240 | -0.01 | | 0.53 | .981 |
| PSA | -1.33 | 0.68 | | 0.056 | 0.00 | 0.89 | | .990 | 0.32 | 0.22 | | .150 | 0.02 | | 0.18 | .920 | -0.58 | | 0.40 | .150 | -0.24 | | 0.51 | .639 |

*Note.* PCQ = Problem complexity questionnaire; CA = Causal analysis; PSA = Problem solving analysis; β = standardized regression coefficient; SE = Standard error.

**Supplementary Section 3**

***Perceived Complexity of Problems Questionnaire***
**Recall any problems you’ve had in the *last 2 weeks*****. Please circle the number that best represents how much you agree with each the following statements:**

|  | ***Strongly Disagree*** | ***Somewhat Disagree*** | ***Somewhat Agree*** | ***Strongly Agree*** |
| --- | --- | --- | --- | --- |
| 1. These problems are complicated. | **1** | **2** | **3** | **4** |
| 1. These problems are difficult to solve. | **1** | **2** | **3** | **4** |
| 1. These problems have left me in a dilemma. | **1** | **2** | **3** | **4** |
| 1. It is difficult to see what would be a good solution to these problems. | **1** | **2** | **3** | **4** |
| 1. I don’t yet know how to resolve these problems. | **1** | **2** | **3** | **4** |
| 1. It is difficult to effectively cope with these problems. | **1** | **2** | **3** | **4** |
| 1. These problems have left me confused about what to do. | **1** | **2** | **3** | **4** |
| 1. I am uncertain about how to deal with these problems. | **1** | **2** | **3** | **4** |

***Psychometric information for the Problem Complexity Questionnaire (PCQ)****Inter-item correlations*PCQ1 (“These problems are complicated.”) (0.66)
PCQ2 (“These problems are difficult to solve.”) (0.71)
PCQ3 (“These problems have left me in a dilemma.”) (0.65)
PCQ4 (“It is difficult to see what would be a good solution to these problems.”) (0.66)
PCQ5 (“I don’t yet know how to resolve these problems.”) (0.70)
PCQ6 (“It is difficult to effectively cope with these problems.”) (0.75)
PCQ7 (“These problems have left me confused about what to do.”) (0.68)
PCQ8 (“I am uncertain about how to deal with these problems.”) (0.74)

*Total item correlations*PCQ1 (0.82)
PCQ2 (0.87)
PCQ3 (0.80)
PCQ4 (0.83)
PCQ5 (0.86)
PCQ6 (0.91)
PCQ7 (0.85)
PCQ8 (0.91)

Total item correlation mean = 0.86

*Associations between problem number and problem complexity (at Week 1) and depression, remission, and problem complexity (at Weeks 5 and 16)*

|  | *Week 5* | | | *Week 16* | |  |
| --- | --- | --- | --- | --- | --- | --- |
|  | Depression | Remission | PC | Depression | Remission | PC |
| *Week 1* | *r_s_ (p)* | *r_s_ (p)* | *r_s_ (p)* | *r_s_ (p)* | *r_s_ (p)* | *r_s_ (p)* |
| PC | 0.11,  *p* = .369 | -0.30,  *p* = .018 | -0.42,  *p* < .001 | 0.32,  *p* = .026 | -0.23,  *p* = .105 | -0.14,  *p* = .332 |
| NUM | 0.13,  *p* = .315 | -0.13,  *p* = .278 | 0.00,  *p* = .989 | 0.16,  *p* = .268 | -0.11,  *p* = .460 | 0.01,  *p* = .960 |

*Note.* PC = Problem complexity; NUM = Problem number. Depression scores were adjusted for baseline depression by subtracting Week 1 depression from Week 5 and Week 16 depression. Problem complexity scores were adjusted for baseline problem complexity by subtracting Week 1 problem complexity from Week 5 and Week 16 problem complexity.
